# Long-term tumor control of spinal dissemination of cerebellar glioblastoma multiforme by combined adjuvant bevacizumab antibody therapy: a case report

**DOI:** 10.1186/1756-0500-7-496

**Published:** 2014-08-07

**Authors:** Thomas Linsenmann, Camelia M Monoranu, Giles H Vince, Thomas Westermaier, Carsten Hagemann, Almuth F Kessler, Ralf-Ingo Ernestus, Mario Löhr

**Affiliations:** Department of Neurosurgery, Julius-Maximilians-University, 97080 Wuerzburg, Germany; Department of Neuropathology, Julius-Maximilians-University, 97080 Wuerzburg, Germany; Department of Neurosurgery, General Hospital, 9020 Klagenfurt, Austria

**Keywords:** Glioblastoma, Spinal dissemination, Bevacizumab, Temozolomide, Irradiation

## Abstract

**Background:**

Glioblastoma multiforme located in the posterior fossa is extremely rare with a frequency up to 3.4%. Compared with glioblastoma of the hemispheres the prognosis of infratentorial glioblastoma seems to be slightly better. Absence of brainstem invasion and low expression rates of epidermal growth factor receptor are described as factors for long-time survival due to the higher radiosensitivity of these tumors.

**Case presentation:**

In this case study, we report a German female patient with an exophytic glioblastoma multiforme arising from the cerebellar tonsil and a secondary spinal manifestation. Furthermore, the tumor showed no O (6)-Methylguanine-DNA methyltransferase promotor-hypermethylation and no isocitrate dehydrogenase 1 mutations. All these signs are accompanied by significantly shorter median overall survival. A long-term tumor control of the spinal metastases was achieved by a combined temozolomide/bevacizumab and irradiation therapy, as part of a standard care administered by the treating physician team.

**Conclusion:**

To our knowledge this is the first published case of a combined cerebellar exophytic glioblastoma with a subsequent solid spinal manifestation. Furthermore this case demonstrates a benefit undergoing this special adjuvant therapy regime in terms of overall survival. Due to the limited overall prognosis of the disease, spinal manifestations of glioma are rarely clinically relevant. The results of our instructive case, however, with a positive effect on both life quality and survival warrant treating future patients in the frame of a prospective clinical study.

## Background

Glioblastoma mulitforme (GBM) is the most common primary tumor of the central nervous system (CNS) comprising approximately 50% of all primary intracranial tumors [1]. It is located most frequently in the cerebral hemispheres and the peak age of onset is the sixth or seventh decade. GBM located in the cerebellum is extremely rare with a frequency of 0% to 3.4% [2–5]. The standard treatment is surgical tumor excision to the extent feasible, followed by radiotherapy and temozolomide chemotherapy [6, 7]. Compared to GBM of the hemispheres, the prognosis of infratentorial GBM seems to be slightly better. Absence of brainstem invasion and a low expression rate of epidermal growth factor receptor (EGFR) are described as factors for long–time survival due to the higher radiosensitivity of these tumors [8, 9].

Extracranial metastasis from GBM is rare, with a reported frequency of only 0.44%. In the reported cases, metastases occurred in regional lymph nodes, lungs, pleura, and occasionally in the bone and liver. Spinal metastases are clinically rarely detected [10]. Spinal cord involvement can be due to a leptomeningeal or vertebral localization, whereas intraparenchymal metastasis of the myelon is extremely rare. Although involvement of the spinal cord has been noted with increasing frequency in recent years, the literature provides 42 documented cases [11].

## Case presentation

**Figure 1:**
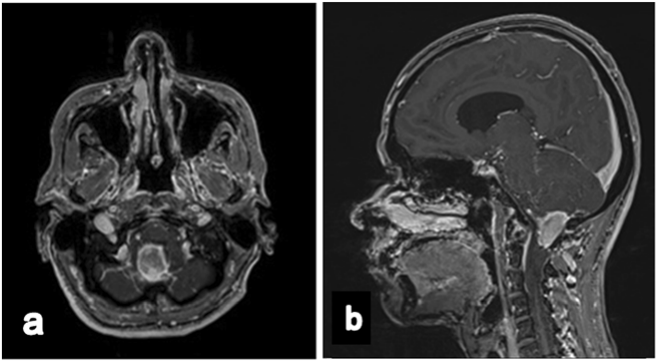
**Preoperative (a) axial and (b) sagittal T1-weighted magnetic resonance imaging after gadopentetate dimeglumine application, demonstrating a well-defined enhancing mass located dorsal from the medulla oblongata with brainstem attachment.**

A 55-year-old German woman presented at our department with a short history of persistent headache, vertigo, nausea, and a generalized seizure. Magnetic resonance imaging (MRI) showed a well-defined tumor approximately 1.7 × 3.7 × 2.6 cm in size, located at the cranio-cervical junction with dorsal compression of the medulla oblongata, and in close relation to the right vertebral artery. The tumor was hyperintense on T2-weighted images and displayed an intense, nearly homogeneous gadolinium (Gd) enhancement. Due to its close contact to the dura, the initial tentative neuroradiological diagnosis was an infratentorial meningioma [Figure 1]. The tumor was completely removed via a median suboccipital craniotomy, opening of the foramen magnum, and resection of the dorsal arch of C1. Microsurgical complete excision of the tumor was achieved by the aid of electrophysiological monitoring (Electromyographies (EMG) of the lower cranial nerves, somatosensory evoked potentials (SEP) and motoric-evoked potentials of the medianus nerve (MEP)).

**Figure 2:**
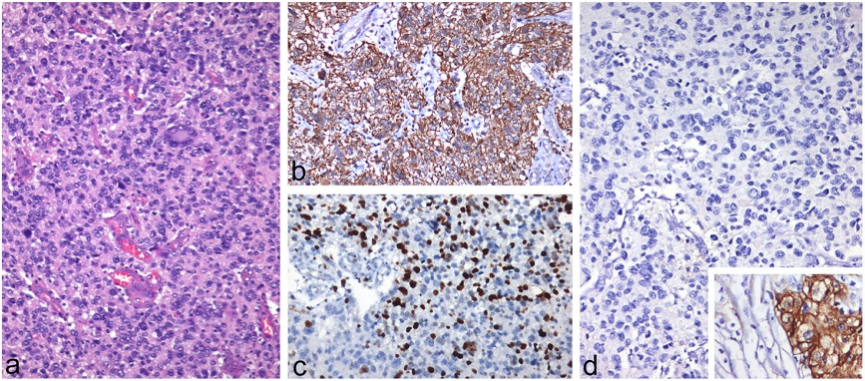
**Histological examination on formalin fixed and paraffin embedded tissue.** **a)** Glioblastoma with high cellular pleomorphism and microvascular proliferation (H&E, x100); **b)** immunohistochemistry for glial fibrillary acidic protein shows strong expression in tumor cells (x100); **c)** high proliferation as determined by the antibody Ki67 (x100); **d)** lack of epidermal growth factor receptor in glioblastoma cells (x100); in higher magnification (x200) colon carcinoma as positive control (inset).

The histological examination on formalin fixed and paraffin embedded tissue confirmed the diagnosis of a glioblastoma multiforme WHO IV with a typical high cellular pleomorphism, microvascular proliferations, and necrotic areas [Figure 2] but without EGFR-expression. Immunhistochemistry also showed a missing MGMT O(6)-Methylguanine-DNA methyltransferase promotor-hypermethylation (MGMT) and no isocitrate dehydrogenase 1 mutation (IDH1).

The postoperative course of the patient was uneventful. She underwent radiochemotherapy with an over-all dose of 54 Gy applied in fractions of 1,8 Gy twice a day over a period of 3 weeks and concomitant temozolomide chemotherapy (75 mg/m^2^ body surface). Adjuvant temozolomide chemotherapy according to the Stupp regimen (200 mg/m^2^ body surface) [8] was administered 12 times. After 15 months without new tumor progression MRIs showed in the area of the right cerebellar tonsil a relapse of about 1.4 × 1.9 × 1.6 cm in size.

She underwent a second operation, but because the tumor had infiltrated the cranial nerves IX and X only a subtotal tumor resection could be performed.

The histological examination confirmed GBM with high mitotic rate and again negative MGMT methylation.

**Figure 3:**
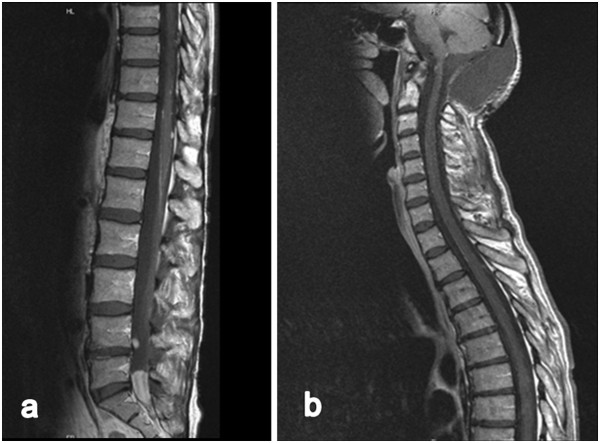
**Sagittal T1-weighted magnetic resonance images enhanced with gadopentetate dimeglumine demonstrating spinal tumor located dorsal of lumbar vertebral body L 4 and a large tumor manifestation dorsal L5 to S1 (a) of approximately 4.0 × 1.2 cm in the sagittal view accompanied by multiple small enhancements in the range of the whole spinal cord (a, b).**

Two weeks later, the patient described lower limb pain, reduced sensitivity, and micturition disorders accompanied by progressive dysphagia. The spinal MRI revealed a small tumor located dorsal to the 4^th^ lumbar vertebral body and a large spinal tumor manifestation dorsal to L5 to S1 of approximately 4.0 × 1.2 cm in the sagittal plane accompanied by multiple gadolinium – enhancing (gd) nodules throughout the whole spinal column [Figure 3]. Diagnosis of a meningeosis carcinomatosa was confirmed by the histological results of a spinal tap.

Radiotherapy was administered to the lumbar spinal mass with an over-all dose of 54 Gy administered in 18 sessions. The cerebellar relapse was treated likewise with 30 Gy in 5 sessions. Irradiation was combined with continuous temozolomide chemotherapy (“one week on, one week off”, 120 mg/day). After therapy the patient presented in an improved overall condition. She still described dysesthesia in the lower limbs but showed a normal function of the bladder and she was also able to walk without aid.

After radio-/chemotherapy the patient consented to off-label intravenous bevacizumab antibody-therapy with 15 mg/kg d1 q21d (according 800 mg bevacizumab) combined with a continuous application of temozolomide 100 mg per day over 25 bouts of therapy, administered by the treating physician team as part of a standard care in case of disseminated GBM.

This procedure is in consistent with the institutional ethics committee instructions according to the Federal Institute for Drugs and Medical Devices. The use is permitted within therapeutic flexibility of the physician in cases of life-threatening diseases without alternative therapeutic option and evidence for therapeutic success (§35bSGBV).

**Figure 4:**
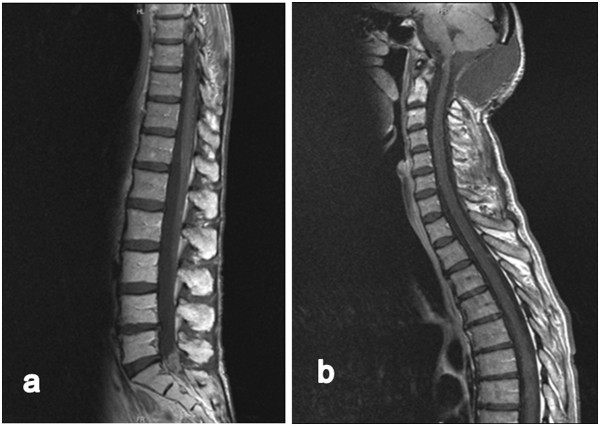
**Sagittal T1-weighted magnetic resonance imaging images enhanced with gadopentetate dimeglumine, demonstrating stable disease without evidence of further tumor growth or new spinal or cranial manifestation 33 months after the first operation and 12 months after spinal irradiation (a, b).**

After 3 months of combined temozolomide/bevacizumab therapy the MRI scans revealed a decrease of cerebellar gd - enhancement and a stable clinical condition concerning the spinal manifestation such as dysesthesia of the lower limbs.33 months after the first operation and 12 months after spinal irradiation the, MRI scans still showed stable disease without evidence of further tumor growth or new CNS manifestations [Figure 4] being aware the fact that it is difficult to discriminate the effects of the RT and the effects of the adjuvant bevacizumab/temozolomide therapy on the spinal cord. The patient presented in a reduced nutritional and general condition with dysphagia, vertigo combined with persistent weakness of the lower limbs, and atypical pneumonia mainly a result of affected caudal cranial nerves, which was not seen in the MRI yet. Well-known side effects under therapy like progressive fatigue and nausea were also observed, but we also realised a good toleration of the therapy in haemograms.

The most recent MRI scan, 38 months after the first operation, revealed a progressive disease with extended meningeosis carcinomatosa around the brainstem and the cervical spinal cord. Because of the poor clinical condition, further chemotherapy and bevacizumab were stopped in favour of best supportive care.

Brain stem and spinal metastases of supratentorial glioblastoma multiforme are well known in the late course of the disease, but are rarely symptomatic. In a hospital series of 600 GBM patients, the incidence of symptomatic spinal metastases was about 2% [12]. To date, the occurrence of an expophytic cerebellar glioblastoma in combination with a solid spinal manifestation has not yet been reported.

Fiorentino *et al.* described combined radiotherapy and bevacizumab in a case of supratentorial GBM with spinal manifestation. This patient died 6 months following diagnosis of metastasis and 3 months after the end of therapy [13]. No specific data have been published yet about treatments with bevacizumab of GBM with spinal manifestation.

A technical enquiry to Hoffmann-La Roche Ltd. Basel Switzerland by the corresponding author revealed that there is no data available concerning the bioavailability of bevacizumab in the cerebrospinal fluid (CSF).

Despite the fact that the prognosis of GBM of the posterior fossa seems to be slightly better than GBM of the cerebral hemispheres, we confirmed in our case an absence of IDH -1 mutation, a negative MGMT methylation, and a meningeosis carcinomatosa as a “signum malum omnis”. Altogether, these are negative predictors of a good response to chemotherapy and long overall survival.

On the other hand, an off-label-use of intravenous bevacizumab combined with temozolomide and radiotherapy arrested a further tumor growth at least for 33 months in the presented case.

## Conclusions

Exophytic cerebellar glioblastoma of the posterior fossa is a rare occurrence. This is also the first report of a secondary spinal manifestation of this entity.

This report highlights the potential of a combined adjuvant therapy with bevacizumab to achieve a long-term tumor control in the case of spinal manifestation of an exophytic glioblastoma multiforme of the posterior fossa.

Due to the limited overall prognosis of the disease, spinal manifestations of glioma are rarely clinically relevant.

This is a single case study, thus we cannot postulate this therapy as standard therapy. The results of our instructive case, however, with a positive effect on both life quality and survival warrant treating future patients in the frame of a prospective clinical study.

## Consent

Written informed consent was obtained from the patient for publication of this case report and accompanying images. A copy of the written consent is available for review by the Editor-in-Chief of this journal.

## Abbreviations

CNS: Central nervous system
CSF: Cerebrospinal fluid
EGFR: Epidermal growth factor receptor
EMA: European Medicines Agency
EMG: Electromyographies
GEFAP: Glial fibrillary acidic protein
GBM: Glioblastoma multiforme
Gd: Gadolinium
Gd-DTPA: Gadopentetate dimeglumine
IDH-1: Isocitrate dehydrogenase 1
MEP: Motoric-evoked potentials
MGMT: O(6)-Methylguanine-DNA methyltransferase
MRI: Magnetic resonance imaging
SEP: Somatosensory evoked potentials.
